# A bibliometric analysis of African dental research and the sustainable development goals, 2016–2023

**DOI:** 10.3389/froh.2024.1498827

**Published:** 2024-11-21

**Authors:** Maha El Tantawi, Ahmed Bhayat, Moréniké Oluwátóyìn Foláyan

**Affiliations:** ^1^ Department of Pediatric Dentistry and Dental Public Health, Faculty of Dentistry, Alexandria University, Alexandria, Egypt; ^2^ Afrone Network, Faculty of Dentistry, Alexandria University, Alexandria, Egypt; ^3^ Oral Health Initiative, Centre for Population Studies, Nigerian Institute of Medical Research, Lagos, Nigeria; ^4^ Department of Preventive Dental Science, Early Childhood Caries Advocacy Group, Winnipeg, MB, Canada; ^5^ Department of Community Dentistry, University of Pretoria, Pretoria, South Africa; ^6^ Department of Child Dental Health, Obafemi Awolowo University, Ile-Ife, Nigeria

**Keywords:** Africa, oral health, dental research, bibliometric analysis, science mapping, sustainable development

## Abstract

**Background:**

The successful implementation of the 2030 sustainable development Agenda in Africa requires active participation from all sectors, including the dental research sector. The aim of this study was to review dental research published by authors affiliated with institutions in African countries from 2016 to 2023, to map papers related to the sustainable development goals (SDGs), and to identify areas of emphasis and gaps in SDGs-related papers on oral health in Africa.

**Methods:**

We conducted a bibliometric analysis of dental literature in Africa (2016–2023) using Scival for performance analysis and VOSviewer for science mapping. The papers addressing and not addressing the SDGs were compared regarding impact, authorship metrics and key phrases. We identified the topic clusters with the greatest number of papers. The key phrase co-occurrence networks and the authors and countries collaboration networks were mapped.

**Results:**

There were 4,173 papers and 622 (14.9%) addressed the SDGs, especially SDG3. SDGs papers had greater impact and were more likely to be published in open access journals than non-SDGs papers. Egypt, Nigeria and South Africa had the greatest number of papers and citations. Four of the top ten authors were females. Most of the top ten journals were open access and only three were in quartile 1 (Q1) journals. Springer and Elsevier were the top publishers. The top research clusters addressed rehabilitative care including implants, endodontics, ceramics and zirconia. SDGs papers, however, addressed oral hygiene in caries prevention and to control systemic diseases. Collaboration networks were affected by geographic location and languages. Dental research in Africa is concentrated in three countries and mainly addresses rehabilitative care. SDGs papers had an impact above the global average and addressed prevention and non-communicable diseases.

**Conclusions:**

There is minimal yet increasing contribution of African countries to the evidence on oral health and the SDGs. The focus is on a limited number of SDGs, and publications are from very few countries in Africa. There is a need to focus oral health-related SDGs research on addressing local problems.

## 1 Introduction

Sustainable development is a comprehensive approach that aims to meet present needs without compromising the ability of future generations to do the same (1). It envisions a future where human societies thrive while maintaining the stability of vital systems (2). It stresses that economic development must be socially inclusive and environmentally sustainable to ensure long-term benefits for people and the planet (3). Sustainable development promotes economic growth that uses resources efficiently and responsibly, fosters innovation, and builds resilient infrastructure (4). Through social sustainability, it focuses on equitable access to resources, opportunities, and services for all to reduce poverty, inequality, and social exclusion and promote well-being, justice, and human rights (5). In addition, it addresses environmental sustainability by protecting natural resources, ecosystems, and biodiversity, to address climate change, pollution, and the ecological footprint of human activities (6).

The United Nations' 2030 Sustainable Development Agenda, adopted in 2015, aims to achieve sustainability. It includes 17 Sustainable Development Goals (SDGs) that address poverty, inequality, climate change, justice and others (7). The agenda ensures that even the most vulnerable populations benefit from development efforts (8). It recognizes the economic, social, and environmental development are linked, stresses fairness in access to resources and opportunities, and promotes caution to prevent irreversible environmental damage (9). By aligning policies with the SDGs, countries can build resilient, sustainable economies, promote responsible business practices, and engage civil society in advocacy and implementation efforts (10).

Africa is home to 40% of the world's population who live on less than $1.90 a day (11). The continent also has the world's youngest population, with a median age of 19 years (12), highlighting the need to invest in education, skills development, and job creation to reduce youth unemployment. Agriculture is the backbone of many African economies. Yet, the continent suffers from food insecurity (13). Also, the continent grapples with significant health challenges, including high rates of infectious diseases, maternal and child mortality, and emerging health threats (14), alongside conflicts, political instability, and weak governance (14). The high burden of oral diseases on the continent is a newly recognized problem that is yet to be addressed (15). The 2030 Agenda offers a roadmap for sustainable development, through partnership to bring in resources and expertise to tackle challenges such as migration, trade issues and climate change.

The successful implementation of the 2030 Agenda in Africa requires strong political will, effective governance, adequate resources, and active participation from all sectors, including governments, civil society, the private sector, and international partners (10). Plans should be based on the science of the environment linked to an understanding of economic, political, and cultural changes (16). Addressing the high burden of oral diseases in Africa also needs to be based on evidence from research. The aim of this study was to review the dental literature by authors affiliated with institutions in African countries from 2016 to 2023, to map papers related to the SDGs, and to identify areas of emphasis and gaps in SDGs-related papers on oral health in Africa.

## 2 Materials and methods

To develop this bibliometric analysis, we used Donthu et al. (17) for guidance. We searched for papers by authors affiliated with African institutions, retrieved them and conducted performance analysis and science mapping. Bibliometric analysis is based on metrics calculated by electronic databases. Compared to Web of Science (WoS), Scopus is likely to include more papers and journals per subject area (18). In 2015, Elsevier published its report about global sustainability and the impact of sustainability-related research based on papers in their Scopus database (19). They have published updated reports since that time and used Scival, their research performance assessment tool, to map SDG research (20). We used Scopus and Scival in this paper.

### 2.1 Literature search

We searched the Scopus database for papers in the field of Dentistry produced by authors from African institutions. We combined terms indicating the subject area of Dentistry with the names of African countries. The search strategy is in Supplementary Table S1.

### 2.2 Eligibility criteria

The inclusion criteria were papers published from January 1st, 2016, after the SDGs were established till December 31st, 2023, the last complete year in Scopus, including only articles and reviews, written in English and published only in journals. Papers published in non-dental journals were excluded. Four duplicates were also excluded.

### 2.3 Data analysis

We exported the search results from Scopus as a comma-separated values (CSV) file. We extracted the Scopus Electronic Identifier number (EID) into a text file and imported this into Scival (21) for performance analysis. The analysis results were exported from Scival (21) as a spreadsheet including the SDGs details. The papers were categorized into those addressing and not addressing SDGs. Separate files were created for each subset of papers, and they were imported again to Scival (21) for performance analysis of the two subsets separately.

The Scopus CSV file was also imported into VOSviewer (22) to create a map of bibliographic data, visualizing co-authorship based on authors and countries and co-occurrence of author keywords.

### 2.4 Performance analysis

All papers, those addressing and those not addressing SDGs were included in the performance analysis based on metrics from Scival (23). We assessed the number of papers and citations per year over the study period. The type of paper (article or review) was identified. The number of authors was counted, and collaboration type was identified, including international (where authors from at least two different countries were listed on the paper), only national (from one country only), only institutional (from same institution), single author as well as academic-corporate or industry collaboration.

We also assessed research impact in terms of the number of cited papers, total citations, citations per paper and field weighted citation impact (FWCI) which is the ratio of citations relative to the expected global average for the subject field, paper type and publication year. The global FWCI is 1.0 (23) and FWCI above 1 indicates above the global average citations and vice versa.

Research impact was also assessed by counting the papers which are among the top 10% most cited papers and the number of papers cited by policy documents. Policy documents are documents written by or for policymakers and included in Overton, which is a database of policy documents. Policy documents include governmental white papers, transcripts of parliament sessions, papers by think tanks, working papers, official reports, and others (24, 25).

We counted the number of papers in the top 10% journals and in journals with different quartiles (Q1–4) with quartiles based on CiteScore percentiles. Q1 journals are the top 25% and the most prestigious (26).

We also counted the number of papers with different publishing models (27): gold open access where papers are published with Creative Commons license and are available at the publisher's website, green open access making available published papers or accepted-for-publication versions at a repository, bronze open access where published papers or accepted-for-publication versions are available at the publisher's website with free access, and non-open access papers published with a traditional publishing model. Also, the number of papers addressing each SDG was counted.

We identified the top 10 most productive authors, whether they were male or female, their affiliation, country, the percentage of papers where they were 1st authors, the number of their papers, citations and FWCI.

We also identified the topmost 10 productive institutions and countries, and the top 10 journals based on number of papers. The number of papers produced by these entities was counted, and the total citations and FWCI were determined.

We identified the 10 most cited papers, whether they were articles or reviews, the year of publication, the number of authors, citations and the affiliation of the first author. Also, we identified the top 10 publishers of the journals where most papers were published.

We extracted topic clusters to analyze the most frequently addressed research topics. These clusters are topics aggregated based on similar research interests that give a broad understanding of the research conducted by an entity (28). The topics that form a research cluster consist of a collection of papers with similar interest representing fields of research (29). To create the topics, an algorithm classifies the papers based on their references lists. Clusters are formed based on pre specified cutoff values of citation link strength. One paper can belong to only one topic and one topic cluster. Scival has about 94,000 topics and 1,500 topic clusters. The topic and topic cluster names are produced in a data-driven way from key phrases obtained from the title, abstract and author keywords of the paper (30).

There are seven topic clusters in the subject area “Dentistry” in Scival: (1) Implant; Orthodontics; Lower Jaw, (2) Scanning Electron Microscopy; Mechanical Strength; Zirconia, (3) Endodontics; Dental Pulp; Lower Jaw, (4) Fluoride; Oral Hygiene; Streptococcus mutans, (5) Oral Hygiene; Gum (Oral Cavity); Diabetes, (6) Temporomandibular Joint; Masticatory Muscle; Thermography and (7) Cleft; Orthodontics; Palate. For each topic cluster, we identified the number of papers, the FWCI and the global prominence percentile. The prominence percentile indicates the momentum of the topic and the possibility of securing funds and research grants (31). The prominence percentile is based on the total citations, the total views and the average CiteScore of the journals in which the papers in the topic cluster are published.

The publication share (PS) was also calculated. This is the percentage of papers in the topic cluster that are produced by the entity, in this case African countries, relative to the global production in the same topic cluster.

Key phrases were also categorized into whether they described (1) diseases or conditions; (2) treatment modality or procedure; (3) intra oral site or location and (4) research methods or study design. The number of papers in which the keywords occurred was divided by the number of papers in each subset (papers addressing and not addressing SDGs) to identify the proportion of the key phrase in each subset. We then subtracted the proportions from each other to differentiate between key phrases in each subset.

### 2.5 Science mapping

We used VOSviewer to visualize the scientific social networks. In the social network map, an entity (author, country or keyword) is represented by a node or a circle and entities within the same cluster or group have the same color. If authors or countries collaborate or keywords co-exist in a paper, an edge or a link/line is drawn between them. The more frequent the collaboration or co-occurrence, the thicker the edge. Only authors or countries with at least five papers and five citations were represented. Only keywords with at least five occurrences were represented. Linlog/modularity normalization was used and attraction of 4 and repulsion of −1 were used to generate the network layout. The node scale was set to correspond to the number of papers so that larger nodes indicate authors or countries with greater number of papers or keywords with greater numbers of papers where they occur.

## 3 Results

The search returned 4,451 papers. We excluded four duplicates and 274 papers from 18 non-dental journals. Thus, 4,173 papers were included in the bibliometric analysis (Supplementary Figure S1).

**Figure 1:**
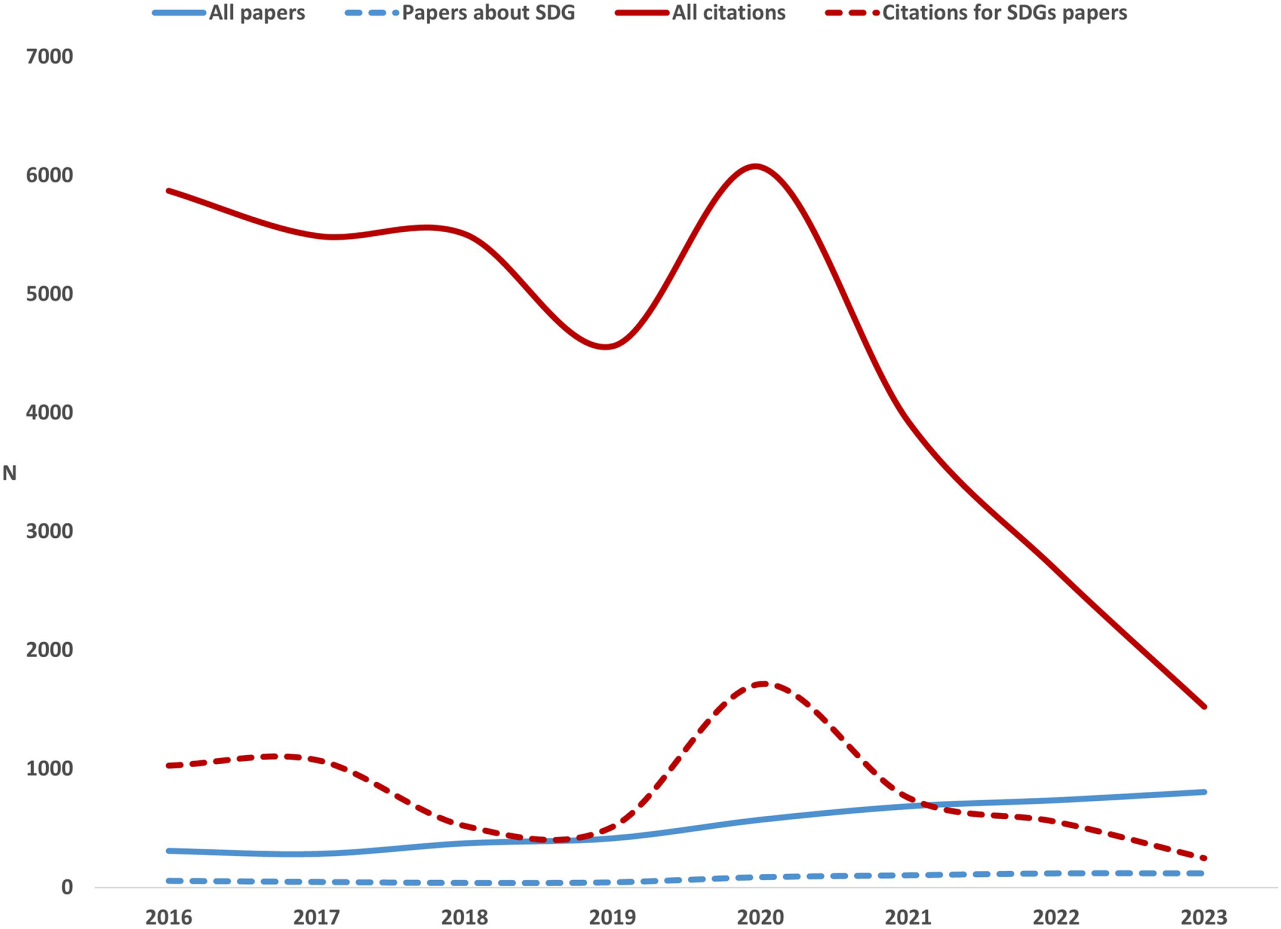
Number of yearly papers and citations, 2016–2023 for all papers and SDGs-papers.

The total number of papers increased steadily from 308 in 2016 to 805 in 2023, percent increase = 161.4% (Figure 1). There were 57 papers focusing on SDGs in 2016, and this peaked at 120 in 2022 and 2023, a percentage increase of 110.5%. The total number of citations for all papers peaked in 2020 at 6,072 and reached 1,525 by 2023. Citations for SDG-related papers peaked at 1,716 in 2020 and declined to 249 by 2023.

Table 1 shows that 622 papers (14.9%) addressed the SDGs and 93% were articles. Only 0.2% were based on academic-corporate collaboration and 3.8% had single authors. Most papers were cited (79.1%), at a level similar to the global average (FWCI = 1.00). Also, 2.2% of the papers were cited by policies, 66.1% of papers were published in Q1 or Q2 journals and 42.1% were published using a non-open access model.

**Table 1:** Performance analysis of all papers, papers addressing and not addressing the SDGs 2016–23.

Performance indicators		All *N* = 4,173	With SDGs *N* = 622	With no SDGs *N* = 3,551
Type	Article: *n* (%)	3,882 (93.0)	564 (90.7)	3,318 (93.4)
	Review: *n* (%)	291 (7.0)	58 (9.3)	233 (6.6)
Collaboration	International collaboration: *n* (%)	1,878 (45.0)	305 (49.0)	1,573 (44.3)
	Only national: *n* (%)	1,191 (28.5)	158 (25.4)	1,033 (29.1)
	Only institutional: *n* (%)	946 (22.7)	132 (21.2)	814 (22.9)
	Single author: *n* (%)	158 (3.8)	27 (4.3)	131 (3.7)
	Academic-corporate collaboration: *n* (%)	10 (0.2)	4 (0.6)	6 (0.2)
Authors	Number of authors	10,517	2,770	8,682
Citations	Yes: *n* (%)	3,300 (79.1)	501 (80.5)	2,552 (78.4)
	No: *n* (%)	874 (20.9)	121 (19.5)	766 (21.6)
	Number of citations	35,632	6,417	29,215
	Citations per paper	8.5	10.3	8.2
	FWCI	1.00	1.15	0.98
	Top 10% most cited: *n* (%)	441 (10.6)	64 (10.3)	377 (10.6)
Impact	Cited by policies: *n* (%)	93 (2.2)	23 (3.7)	70 (2.0)
Journal quartile	Top 10% journals: *n* (%)	480 (12.6)	82 (14.2)	405 (12.5)
	Q1: *n* (%)	1,214 (31.8)	201 (34.7)	1,021 (31.5)
	Q2: *n* (%)	1,312 (34.3)	219 (37.8)	1,095 (33.8)
	Q3: *n* (%)	938 (24.6)	113 (19.5)	817 (25.2)
	Q4: *n* (%)	356 (9.3)	46 (7.9)	309 (9.5)
Access model	Non-open: *n* (%)	1,757 (42.1)	211 (33.9)	1,546 (43.5)
	Open, gold: *n* (%)	1,613 (38.7)	269 (43.2)	1,344 (37.9)
	Open, green: *n* (%)	1,075 (25.8)	201 (32.3)	874 (24.6)
	Open, bronze: *n* (%)	197 (4.7)	33 (5.3)	164 (4.6)

Papers which addressed the SDGs were more likely than those not addressing the SDGs to be reviews, have international collaboration, be based on academic-corporate collaboration, have greater number of average citations, greater FWCI, be cited by policies, be published in Q1 and Q2 journal and use an open access model.

**Figure 2:**
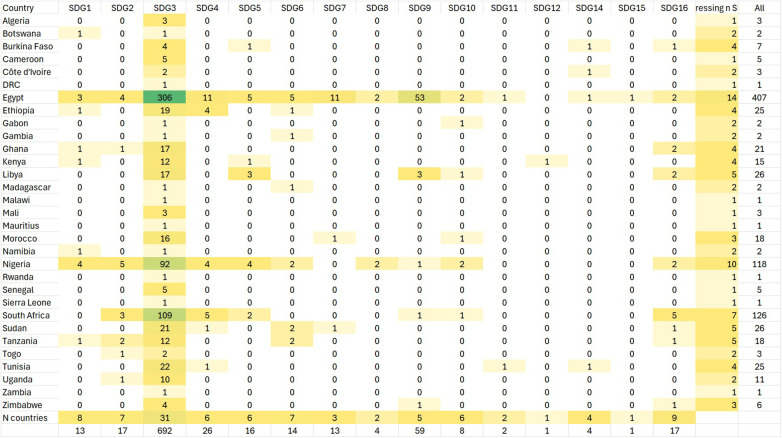
African countries addressing the SDGs.

Most papers addressing SDGs addressed SDG3 (Good health and wellbeing) followed by SDG9 (Industry, innovation and infrastructure, Figure 2). No papers addressed SDG13. Papers from 31 countries addressed the SDGs and all of them addressed SDG3, representing 56.4% of all African Union member states (32). Egypt (*n* = 14), Nigeria (*n* = 10) and South Africa (*n* = 7) addressed the greatest number of SDGs. Only Kenya addressed SDG12 and only Egypt addressed SDG15. Egypt had the greatest number of papers in SDG3 to SDG7 and SDG9. Nigeria had the greatest number of papers addressing SDG1 and 2. South Africa had the greatest number of papers addressing SDG16.

Table 2 shows that among the 10 most productive authors, four were females, including the top two (El Tantawi and Folayan). Seven were affiliated with Egyptian institutions, two with Nigerian institutions and one with a South African institution. Folayan and Elsyad were first authors in >50% of their papers. Elsaka was last author in more than 50% of her papers. Five authors were first or last authors in ≥0% of their papers. Three authors (Adeyemo, Fayed and Abu-Seida) had no papers addressing SDGs (Supplementary Table S2).

**Table 2:** The top 10 most productive authors with African institutions affiliation.

Author (M/F)	Affiliation	1st author (%)	Last author (%)	Policy impact	Docs	Citations	FWCI
1. El Tantawi, Maha (F)	Alexandria University, Alexandria, Egypt	15.2	34.8	7	66	1,517	1.72
2. Folayan, Morenike O (F)	Obafemi Awolowo University, Nigeria	51.2	10.7	2	65	1,207	1.89
3. Elsyad, Moustafa (M)	Mansoura University Mansoura, Egypt	69.8	11.3	2	47	675	1.52
4. Adeyemo, Wasiu L. (M)	University of Lagos, Lagos, Nigeria	4.7	41.9	3	40	367	1.04
5. Fawzy, Karim (M)	Cairo University, Egypt	30.3	42.4	0	30	646	1.75
6. Saber, Shehabeldin (M)	The British University in Egypt, Egypt	37.5	12.5	0	29	244	2.07
7. Elsaka, Shaymaa (F)	Mansoura University Mansoura, Egypt	27.0	51.4	1	28	913	2.71
8. van Heerden, Willie F.P. (M)	University of Pretoria, South Africa	0	40.6	0	26	127	0.80
9. Fayed, Mona (F)	Cairo University, Egypt	0	7.1	2	25	618	1.81
10. Abu-Seida, Ashraf M. (M)	Cairo University, Egypt	3.9	26.9	0	24	241	1.91

Eight of the top 10 most productive institutions were in Egypt, one was in South Africa, and one was in Nigeria (Table 3). Nine of these institutions had FWCI >1. These ten institutions produced 62.5% of all SDGs papers on the continent and had 90.9% of SDGs-related citations (Supplementary Table S2). Most institutions had greater FWCI for papers addressing SDGs than non-SDGs papers (Supplementary Table S2).

**Table 3:** The top 10 entities with the greatest number of papers 2016–2023.

Entity	Number of papers	Citations	FWCI
African institutions			
1. Cairo University (Egypt)	698	6,795	1.03
2. Alexandria University (Egypt)	492	4,722	1.33
3. Ain Shams University (Egypt)	483	3,338	0.85
4. Mansoura University (Egypt)	422	4,146	1.21
5. Al-Azhar University (Egypt)	241	1,972	1.16
6. University of Pretoria (South Africa)	139	1,355	1.11
7. Tanta University (Egypt)	136	927	1.03
8. Obafemi Awolowo University (Nigeria)	127	2,493	1.55
9. National Research Center (Egypt)	108	1,038	1.08
10. Suez Canal University (Egypt)	107	919	1.15
African countries			
1. Egypt	2,732	22,798	1.08
2. South Africa	382	3,743	1.01
3. Nigeria	372	5,278	1.19
4. Tunisia	145	652	0.50
5. Morocco	135	798	0.67
6. Sudan	123	793	0.67
7. Libya	78	1,214	1.51
8. Ethiopia	61	2,342	2.41
9. Ghana	54	987	1.23
10. Kenya	48	321	0.86
Journals (2023 WoS Q)			
1. BMC Oral Health* (Q1)	352	2,437	1.24
2. Ain Shams Dental Journal (Egypt)* (-)	215	58	0.06
3. Journal of Contemporary Dental Practice (-)	110	335	0.39
4. Journal of Prosthetic Dentistry (Q1)	109	1,180	1.38
5. International Journal of Dentistry* (Q2)	102	690	0.75
6. Brazilian Dental Science* (-)	99	197	0.33
7. Clinical Oral Investigations (Q1)	96	984	2.11
8. Saudi Dental Journal* (Q3)	68	601	0.92
9. Journal of International Oral Health (Q4)	66	80	0.22
10. Case Reports in Dentistry* (Q4)	59	193	0.40

* Open access.

The top three most productive countries were Egypt, South Africa and Nigeria (Table 3), producing 83.5% of all papers, with 89.3% of all citations and FWCI >1. The three countries produced 81.5% of the SDGs-papers with 90.9% of the related citations (Supplementary Table S2).

Six of the top 10 journals with the greatest number of papers were open access. Three had no quartiles and had low FWCI (<0.40, Table 3). Conversely, three journals were Q1 with FWCI >1, overall and for papers addressing and not addressing SDGs (Supplementary Table S2). Only BMC Oral Health uses an open access publishing model among the three Q1 journals.

Scival analysis of papers in BMC Oral Health from 2021 to 2023 showed that the number of papers increased by 355% for Egypt, decreased by 66.7% for Nigeria and increased by 150% for South Africa with a total of 252, 55 and 23 papers in these three years respectively.

Springer was the publisher of 22.3% of all papers, followed by Elsevier (21.9%) then Wiley (9.6%) (Supplementary Figure S2).

The top 10 most cited papers included four reviews and three papers that addressed SDG3, two about oral cancers and one about the link between periodontal disease and diabetes (Supplementary Table S4). One paper, a review, had a single author. Four papers had first authors from Ethiopia, Nigeria, Libya and South Africa. The first authors in six papers were affiliated with Egyptian institutions.

Implant & Orthodontics was the topic cluster with the greatest number of papers. It covered developing dental implant surfaces to improve osseointegration (Supplementary Table S3). Next was Scanning Electron Microscopy, Mechanical Strength & Zirconia addressing dental materials to improve restoration durability; then Endodontics & Dental Pulp addressing root canal treatment and stem cell research. These three topic clusters also represented the greatest PS by African countries (1.42%–1.82%). The greatest number of papers were in topic clusters with the greatest prominence percentile.

The greatest number of SDGs-papers were about oral hygiene in relation to fluoride and its impact on caries, or in relation to gum disease and its link with systemic diseases. The oral hygiene-related clusters had the greatest percentage of PS with FWCI >1. In each topic cluster, SDGs papers had greater FWCI than non-SDGs papers except the cluster of endodontics and dental pulp (Supplementary Table S3).

The most frequent keyword in the diseases/conditions category was dental caries then edentulism (Supplementary Figure S3). Implants was by far the most frequent keyword in the treatment category. The lower jaw was frequent in the location category. In the methods/design category, the most frequent keyword was *in vitro* studies, cross-sectional and prevalence, and the least frequent were randomized clinical trials and cephalometry.

**Figure 3:**
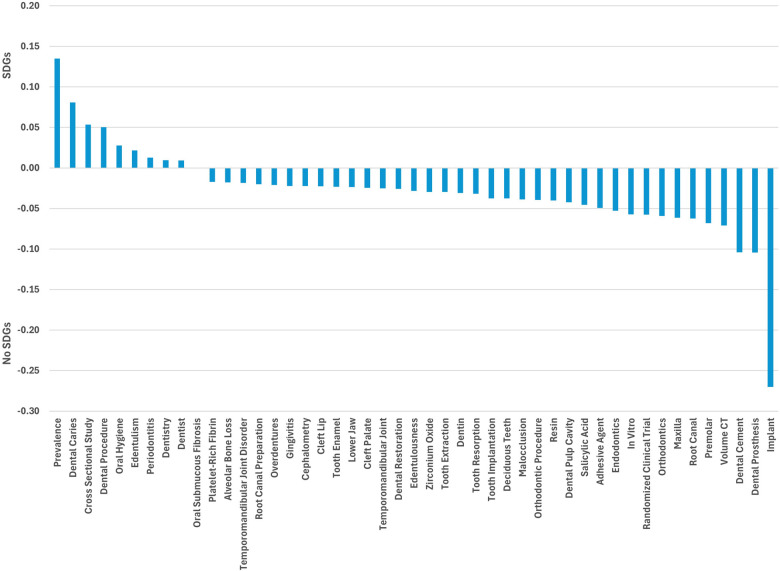
Proportion of top frequent 50 keywords in SDGs-papers and non-SDGs papers (negative sign on *y*-axis indicates greater proportion in non-SDGs papers).

Keywords like prevalence, dental caries, cross-sectional studies, dental procedures and oral hygiene were more frequent in SDGs papers (Figure 3), while implants, dental prosthesis and dental cement were more common in non-SDG papers.

### 3.1 Science mapping

**Figure 4:**
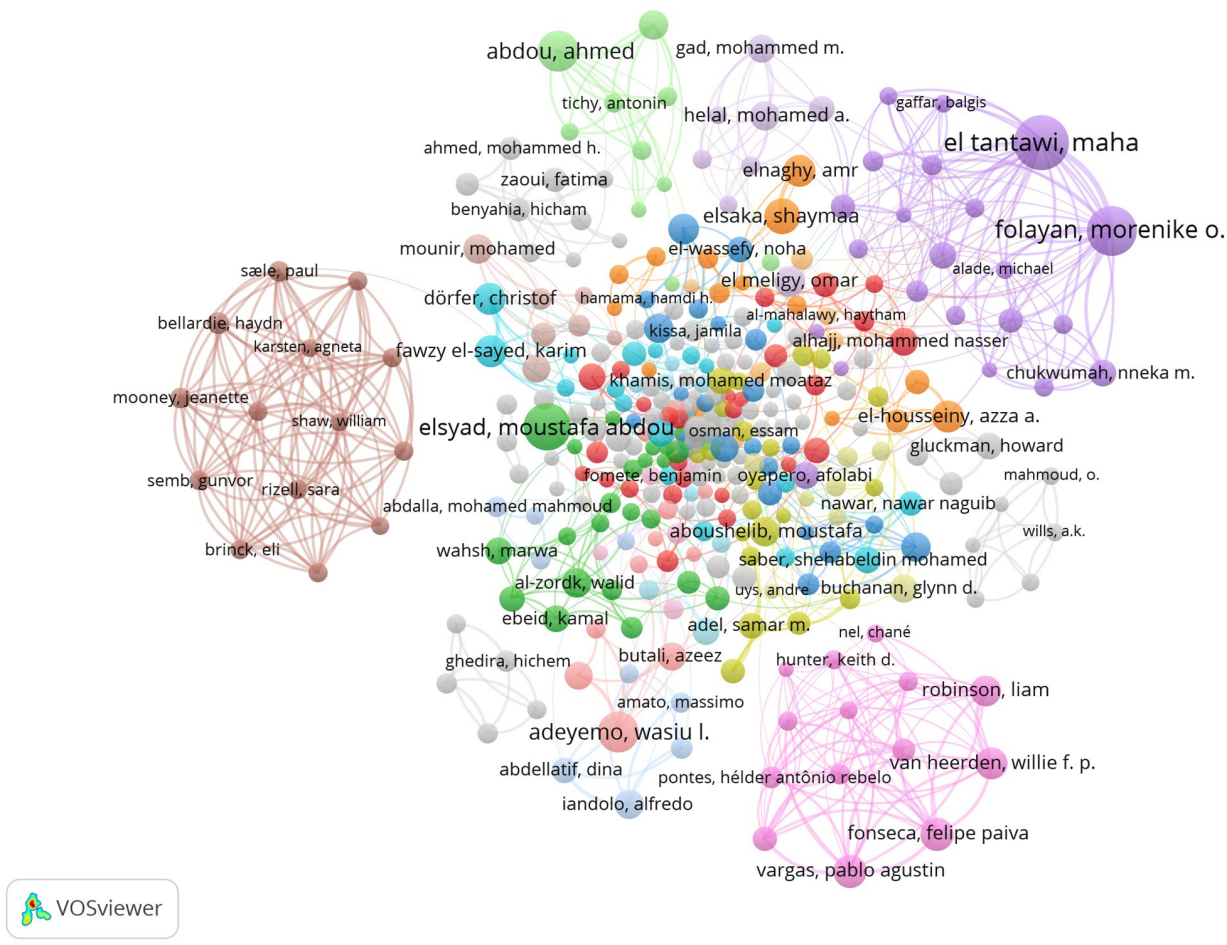
Authors’ co-authorship network map.

There were 403 authors with 5+ papers and citations (Figure 4), including 226 authors connected to others creating 106 clusters with 790 links. The largest cluster included 27 authors (red circles), mostly not affiliated with African institutions. The second cluster included 23 authors (green circles), a mix of authors from North Africa and authors not affiliated with African institutions. The third (blue circles) and fourth (greenish yellow circles) clusters included 23 authors each, mostly Egyptian authors. The top two most productive authors, El Tantawi and Folayan, were in the 5th cluster (purple circles) with 22 authors from Africa and other countries.

**Figure 5:**
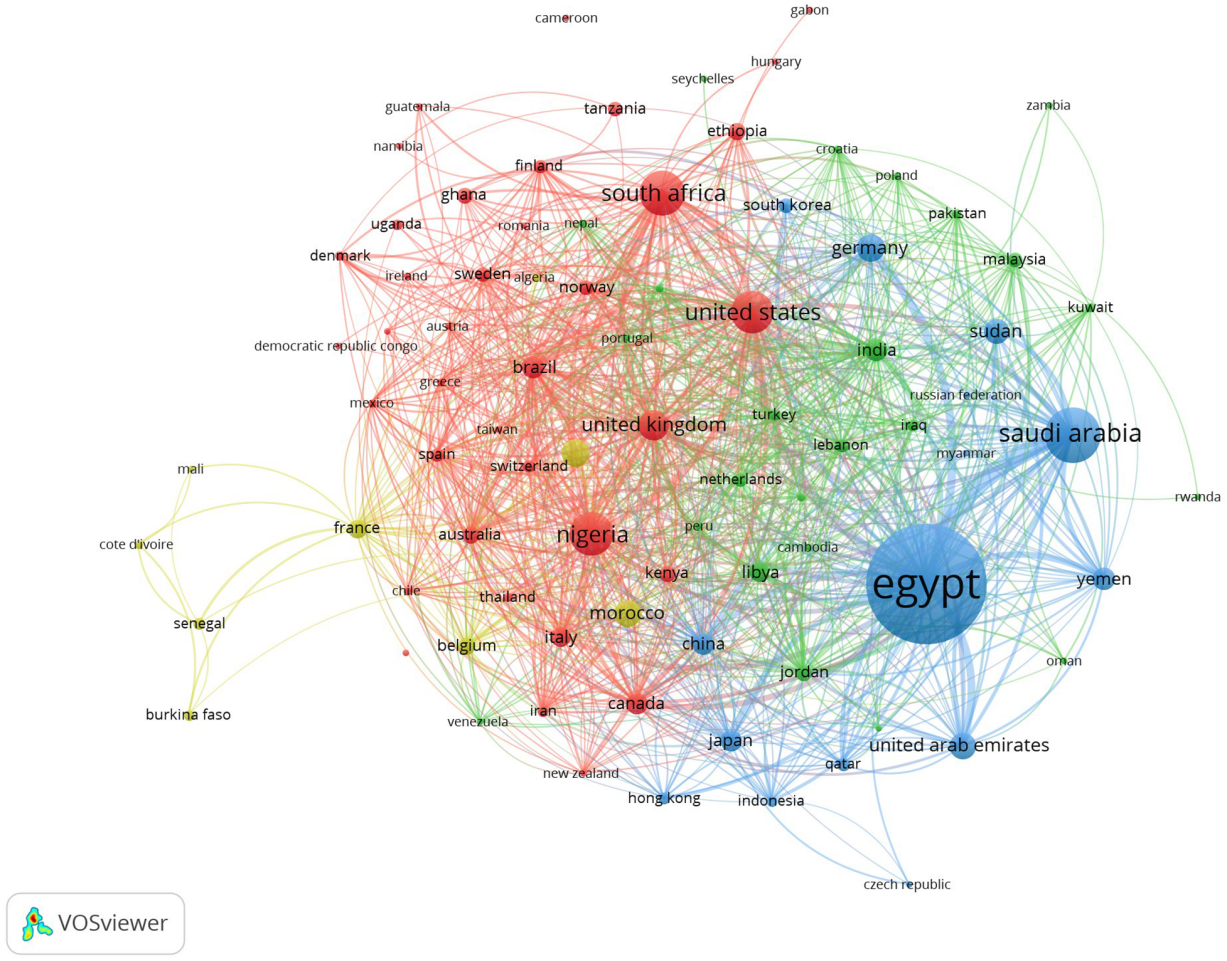
Countries’ co-authorship network map.

There were authors from 169 countries (Figure 5), including 85 countries with 5+ papers and citations. There were four distinct clusters of countries with 1,492 links. The first cluster (red circles) included South Africa, Nigeria, the USA, UK, Brazil among 37 countries. The second cluster (green circle) included India, Malaysia, Jordan, Libya and Tanzania among 24 countries. The third clusters (blue circles) included Egypt, Saudi Arabia, Germany, Sudan among 15 countries. The fourth cluster (yellow circle) included Algeria, Cote d'Ivoire, Burkina Faso, Mali, Morocco, Senegal, Tunisia, Belgium and France with a total of nine countries.

**Figure 6:**
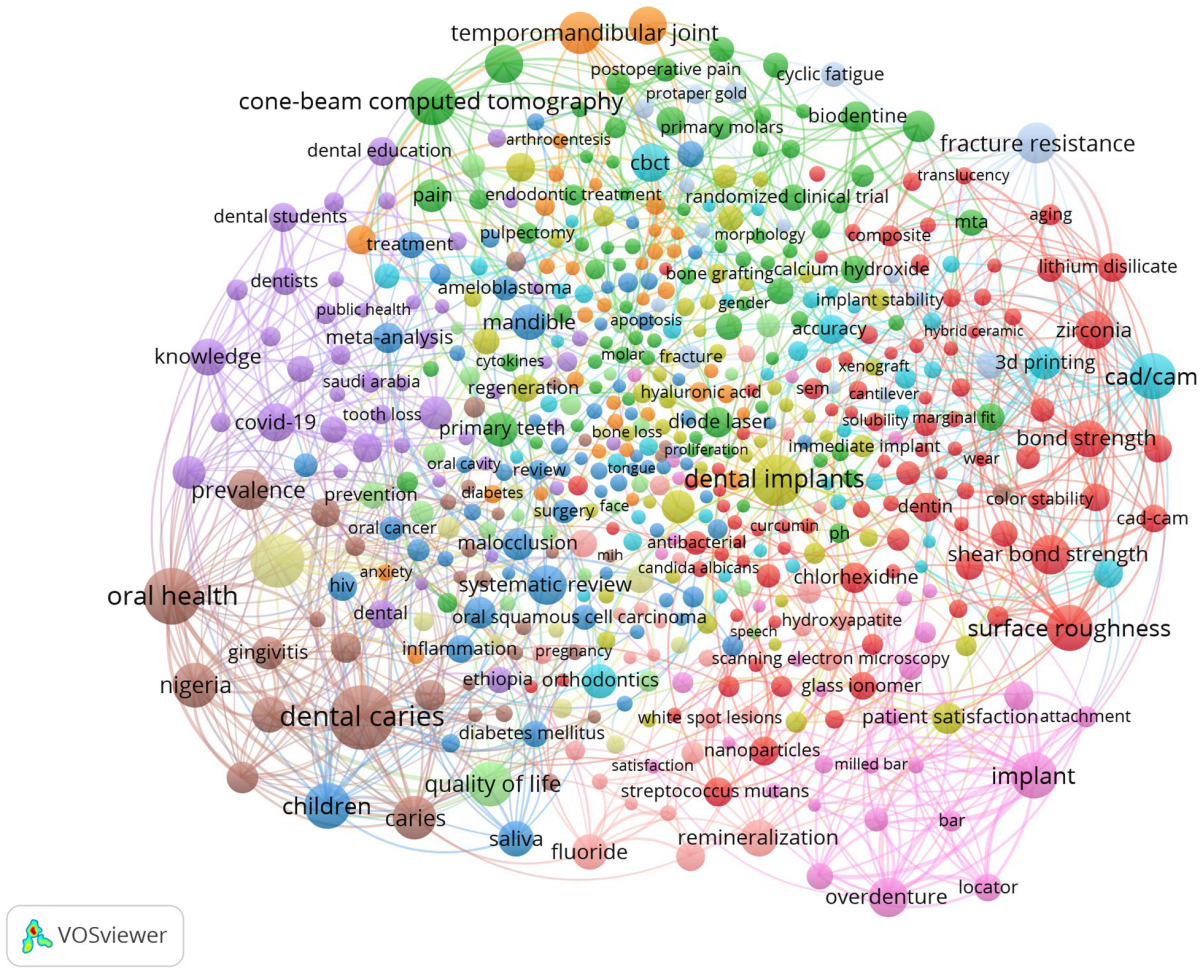
Author keywords co-occurrence network map.

There were 8,847 author keywords in the co-occurrence network (Figure 6), including 556 with 5+ occurrences and 555 connected to other keywords. There were eight clusters with 4,041 links. The first cluster (red circles) had 118 keywords including periodontitis, dental implants, saliva, and systematic reviews. The second cluster (green circles) had 110 keywords including oral health, dental caries, quality of life, and malocclusion. The third cluster (blue circle) had 97 keywords including remineralization, biodentin, diode laser, and scanning electron microscope. The fourth cluster (yellow circles) had 94 keywords including CAD/CAM, Zirconia, surface roughness, shear bond strength, and fracture resistance. The remaining clusters had keywords ranging from 1 to 73.

## 4 Discussion

This bibliometric analysis showed that one in seven dental papers by authors from African institutions addressed the SDGs. Compared to non-SDGs papers, papers addressing the SDGs showed more international and corporate collaboration, greater impact and were more accessible. Most papers addressing SDGs focused on SDG3 and, to a lesser extent, SDG9. Egypt, South Africa and Nigeria produced over 80% of the papers from Africa, and researchers and institutions from these countries contributed the greatest number of papers and citations. Four women were among the top 10 productive authors. The Q1 journals where most papers were published were BMC Oral Health, Journal of Prosthetic Dentistry and Clinical Oral Investigations. BMC Oral Health had the greatest number of SDGs-papers by authors from Africa, and the greatest citations. Springer and Elsevier published most dental papers from Africa. Half the papers were about implants, zirconia, ceramics and endodontics, and these papers did not address the SDGs. The SDGs-papers focused on oral hygiene in relation to caries prevention and the oral-systemic link. The co-authorship networks showed distinct collaboration patterns affected by geographical location and language, where countries in North Africa collaborated with neighboring countries in the Middle East and French speaking African countries collaborated with France and Belgium.

This is the first comprehensive analysis of dental publications on the SDGs in Africa. It utilizes sophisticated bibliometric tools to enhance robustness and reliability and provides valuable insights into the scientific social networks in the field. By focusing on SDG-related research, the study highlights the alignment of dental research in Africa with global sustainability goals, which is crucial to drive policy and funding decisions. The gaps in SDG research can inform research and grants priorities in the continent.

The study, however, has some limitations. It relied on Scopus only to retrieve papers, which might miss some contributions to the field such as papers in journals that are non-Scopus indexed. The inclusion of papers written in English only may introduce a language bias. The limited time span reduced the possibility to identify trends across time. However, the study period followed the SDGs establishment and offered an insight about the responsiveness of dental research in Africa to the SDGs halfway to when the SDGs targets should be achieved. Lastly, we used Scival mapping of papers to the SDGs which is less subject to bias and more reproducible. However, assessing the comprehensiveness of this mapping is beyond the study aims. Despite the study limitations the study has some important findings.

First, dental research in Africa in general, including that which is related to the SDGs, has low productivity and heavy skewness to three of the 55 African Union member states. There are 160 dental schools in Africa including 20.6%% in Egypt, 10.6% in Nigeria and 6.9% in South Africa (33). Evidence shows that information about oral health in Africa is concentrated in countries with dental schools (34), probably because for the researchers in these schools, promotion would be a motive for research productivity. The skewed distribution of research output among African countries calls for concerted, continent-wide efforts for capacity building and networking to empower and mentor researchers with limited access to support from research communities in their own countries.

The low research productivity may also relate to the low priority given to oral health by policy makers and the public (35), the poor capacity and scarce funding (36). Only recently has there been an increase in interest in oral health in Africa with the Global Oral Health Status Report raising concerns about the huge burden of oral diseases in the continent (35). This interest may increase dental research funding. However, there is a risk that, despite this new interest, dental research about Africa may be driven by researchers from the global north because they have better funding and competency to conduct research.

Part of the research funding is needed to support open access publishing. Open access papers can be accessed by readers free of charge (37). Yet, the article processing charges (APCs) of gold open access are paid by authors. Some publishers waive APCs for authors from low- and middle-income countries. Springer, the top publisher for African dental research in the study period, has a country-tiered APC pricing system for 64 journals (38, 39). Elsevier grants full or partial waivers to authors from 126 countries (40) and Wiley to 128 countries (41), depending on country income level. By contrast, Wolters Kluwer provides waivers for authors from 32 countries only (42). Thus, publishers differ in the support they provide for authors from low- and middle-income countries and this may affect publishing in gold open access journals by authors from Africa. On the other hand, Egypt and South Africa have transformative agreements with Springer Nature, the publisher of BMC Oral Health, to pay APCs. This may explain the sharp increase in the number of papers from Egypt in BMC Oral Health after the agreement in 2022. The full effect in South Africa remains to be seen since the agreement has just been signed in 2023. By contrast, the number of papers from Nigeria, where there is no agreement, has sharply decreased and is overall small. Botswana and Namibia have also just signed this agreement (43) and should be monitored for future trends.

Second, dental research in Africa follows global research interests as indicated by the prominence percentile of topic clusters focusing on rehabilitative care such as implants, ceramics and endodontics. The extent to which these topics match the oral health needs of people in Africa is, however, debatable. Africa is home to a greater percentage of young (<15 years old) than old (65+ years old) people (44). Also, 84% of African countries are low or lower middle-income countries (45) with limited ability to pay for costly dental procedures. There is a scarcity of dental workforce (46) and people in Africa spend less than 1 United States Dollar per capita on dental care with expenditure mostly on basic oral healthcare (47). This raises questions about how these complex rehabilitative procedures could have research priority in the continent.

The focus on costly and complex rehabilitative care suggests a colonization of research agenda in Africa where researchers in Africa respond to the prevailing norms in the scientific community, largely dominated by the global North (48). This research focus may increase access to grants and publishing in international journal that are otherwise prejudiced against manuscripts identified as “more suitable to regional journals”. The problem is compounded by gate keeping and the power structures that assign the ability to define what is important to editors and researchers from the Global North. Research decolonization begins by empowering researchers from Africa, increasing their representation in editorial boards of international journals, inviting them as reviewers and working with international collaborators as allies to empower researchers from African countries to focus on their community problems. Also, it is important to develop national oral health research agendas to support oral health policies. These agendas and policies need to be developed based on consensus among stakeholders including community representatives to define problems, identify resources, address the disease burden in Africa and holistically align with the SDGs.

By contrast, the papers that address the SDGs focus on the prevention of oral diseases and their relationship with systemic diseases responding to the urgent need to reduce the burden of oral diseases in Africa (49). In addition, the SDGs papers address universal health coverage and access to essential oral healthcare to reduce the costly dental care that is paid out-of-pocket in the absence of dental health insurance in most African countries (47). The study findings highlight the need to define research priorities to address the oral health problems in Africa and identify context-specific strategies to address them effectively. These problems need to be defined and solved by researchers in Africa using resources mobilized domestically (50).

Third, the positive gender balance among the top researchers in Africa is encouraging and reflects gender equality in a field traditionally dominated by men (51). A balanced gender representation brings diverse perspectives to research, enhancing its quality and scope. The visibility of women in leadership roles is a powerful inspiration for aspiring female scientists, helping to sustain and advance gender equality in oral health research. Additionally, the presence of men and women, as first or last authors, fosters a collaborative and inclusive research culture. This agrees with earlier findings of gender balance reported in Nigeria (52). The findings may reflect the impact of research training programs that address the unique challenges facing women in the field (53) and may also possibly reflect the changing demographics of university applicants by time. However, reliable data about the profile of applicants and newly admitted students in African higher education institutions is scarce. However, more analysis is needed to address context-specific challenges to gender equality in research in Africa.

Fourth, the SDGs papers focused on SDG 3 (Good Health and Well-being) in Egypt, Nigeria, and South Africa. SDG 1 (No Poverty), SDG 2 (Zero Hunger), SDG 5 (Gender Equality), SDG 8 (Decent Work and Economic Growth), SDG 13 (Climate Action) and SDG 16 (Peace, Justice, and Strong Institutions) received less to no attention although they are critical for Africa. The present study shows that SDG3 was the most researched SDG by authors from Africa. This agrees with an earlier bibliometric analysis covering the period 2015–2019 that reported that publications about SDG3 represented the highest percentage (14.2%) of all SDGs-related publication from Africa. However, in terms of absolute numbers, they were less than the number of SDG3-papers from the Americas and Europe (54). Several studies have highlighted the plausibility of links between the SDGs and Oral Health (55–58). These studies highlight the successes and gaps in oral health contribution and response to the SDGs. These are strategic actions that researchers in Africa need to take to ensure that the voice of the continent is represented in global decision-making. Most SDGs papers had high citations which supports our postulation that these papers carry the voice of Africa to global decision-making spaces. A paradigm shift is also needed in oral health research for a wider and more holistic approach that recognizes the interdependence between oral health and the general health and wellbeing of populations. This must acknowledge that the SDGs themselves are interdependent (59). Thus, research on oral health and the SDGs must comprehensively address oral health by targeting sustainable solutions to oral diseases.

In conclusion, this study highlights the minimal yet increasing contribution of African countries to the evidence on oral health and the SDGs for policy making. SDGs papers focused on the SDG3, and were mainly produced by researchers from Egypt, Nigeria and South Africa with variation across other countries in the commitment to sustainable development. The journey to fully achieve the SDGs remains long, with challenges that need to be addressed to promote oral health in Africa. It is important to focus on oral health-related SDGs research that addresses local problems. By building on strengths and addressing limitations to oral health research in Africa, researchers can pave the way for a more sustainable and equitable access to oral health in the continent.

## Data Availability

Publicly available datasets were analyzed in this study. This data can be found here: The data is in the Scopus and Scival databases.
