# Supplementary material for: A bibliometric analysis of African dental research and the sustainable development goals, 2016–2023
Source: Front Oral Health. 2024 Nov 21;5:1498827. doi: 10.3389/froh.2024.1498827 (PMC11617509; doi:10.3389/froh.2024.1498827)
Supplement: Supplementary file 1 [file Table1.docx]

Supplementary Material

# Supplementary Tables

- 1. **Supplementary Table 1.** SCOPUS search strategy

| SUBJAREA ( dent ) AND PUBYEAR > 2015 AND PUBYEAR < 2024 AND ( DOCTYPE ( ar ) OR DOCTYPE ( re ) ) AND ( AFFILCOUNTRY ( burundi ) OR AFFILCOUNTRY ( cameroon ) OR AFFILCOUNTRY ( central AND african AND republic ) OR AFFILCOUNTRY ( chad ) OR AFFILCOUNTRY ( congo AND republic ) OR AFFILCOUNTRY ( dr AND congo ) OR AFFILCOUNTRY ( equatorial AND guinea ) OR AFFILCOUNTRY ( gabon ) OR AFFILCOUNTRY ( s&#227;o AND tom&#233; AND pr&#237;ncipe ) OR AFFILCOUNTRY ( comoros ) OR AFFILCOUNTRY ( djibouti ) OR AFFILCOUNTRY ( eritrea ) OR AFFILCOUNTRY ( ethiopia ) OR AFFILCOUNTRY ( kenya ) OR AFFILCOUNTRY ( madagascar ) OR AFFILCOUNTRY ( mauritius ) OR AFFILCOUNTRY ( rwanda ) OR AFFILCOUNTRY ( seychelles ) OR AFFILCOUNTRY ( somalia ) OR AFFILCOUNTRY ( south AND sudan ) OR AFFILCOUNTRY ( sudan ) OR AFFILCOUNTRY ( tanzania ) OR AFFILCOUNTRY ( uganda ) OR AFFILCOUNTRY ( algeria ) OR AFFILCOUNTRY ( egypt ) OR AFFILCOUNTRY ( libya ) OR AFFILCOUNTRY ( mauritania ) OR AFFILCOUNTRY ( morocco ) OR AFFILCOUNTRY ( sahrawi AND republic ) OR AFFILCOUNTRY ( tunisia ) OR AFFILCOUNTRY ( angola ) OR AFFILCOUNTRY ( botswana ) OR AFFILCOUNTRY ( eswatini ) OR AFFILCOUNTRY ( swaziland ) OR AFFILCOUNTRY ( lesotho ) OR AFFILCOUNTRY ( malawi ) OR AFFILCOUNTRY ( mozambique ) OR AFFILCOUNTRY ( namibia ) OR AFFILCOUNTRY ( south AND africa ) OR AFFILCOUNTRY ( zambia ) OR AFFILCOUNTRY ( zimbabwe ) OR AFFILCOUNTRY ( benin ) OR AFFILCOUNTRY ( burkina AND faso ) OR AFFILCOUNTRY ( cabo AND verde ) OR AFFILCOUNTRY ( cape AND verde ) OR AFFILCOUNTRY ( c&#244;te AND d&apos;ivoire ) OR AFFILCOUNTRY ( ivory AND cost ) OR AFFILCOUNTRY ( gambia ) OR AFFILCOUNTRY ( ghana ) OR AFFILCOUNTRY ( guinea ) OR AFFILCOUNTRY ( guinea-bissau ) OR AFFILCOUNTRY ( liberia ) OR AFFILCOUNTRY ( mali ) OR AFFILCOUNTRY ( niger ) OR AFFILCOUNTRY ( nigeria ) OR AFFILCOUNTRY ( senegal ) OR AFFILCOUNTRY ( sierra AND leone ) OR AFFILCOUNTRY ( togo ) ) AND ( LIMIT-TO ( LANGUAGE , "English" ) ) AND ( LIMIT-TO ( SRCTYPE , "j" ) ) |
| --- |

- 1. **Supplementary Table 2.** Differences between papers addressing and not addressing SDGs in the number of papers and impact by authors, institutions, countries, and journals

| Entity | With SDGs | | | With no SDGs | | |
| --- | --- | --- | --- | --- | --- | --- |
|  | Papers | Citations | FWCI | Papers | Citations | FWCI |
| Author | | | | | | |
| 1. El Tantawi, Maha | 21 | 867 | 2.83 | 45 | 290 | 1.30 |
| 1. Folayan, Morenike O | 24 | 852 | 2.46 | 41 | 355 | 1.02 |
| 1. Elsyad, Moustafa | 4 | 85 | 2.21 | 43 | 590 | 1.34 |
| 1. Adeyemo, Wasiu L | - | - | - | 38 | 362 | 1.02 |
| 1. Fawzy, Karim | 2 | 46 | 1.04 | 28 | 600 | 1.84 |
| 1. Saber, Shehabeldin | 3 | 12 | 0.92 | 26 | 232 | 1.77 |
| 1. Elsaka, Shaymaa | 3 | 15 | 1.19 | 25 | 898 | 2.665 |
| 1. van Heerden, Willie F.P. | 13 | 54 | 0.76 | 13 | 73 | 0.90 |
| 1. Fayed, Mona | - | - | - | 25 | 618 | 1.96 |
| 1. 10. Abu-Seida, Ashraf | - | - | - | 23 | 233 | 1.60 |
| Institutions | | | | | | |
| 1. Cairo University, Egypt | 63 | 733 | 1.19 | 635 | 6,062 | 1.01 |
| 1. Alexandria University, Egypt | 72 | 1,273 | 1.71 | 420 | 3,449 | 1.27 |
| 1. Ain Shams University, Egypt | 52 | 1,099 | 1.28 | 431 | 2,239 | 0.80 |
| 1. Mansoura University, Egypt | 48 | 476 | 1.24 | 374 | 3,670 | 1.20 |
| 1. Al-Azhar University, Egypt | 27 | 521 | 2.79 | 214 | 1,451 | 0.95 |
| 1. University of Pretoria, South Africa | 45 | 356 | 0.90 | 94 | 999 | 1.22 |
| 1. Tanta University, Egypt | 20 | 122 | 0.99 | 116 | 805 | 1.04 |
| 1. Obafemi Awolowo University, Nigeria | 41 | 1,068 | 1.93 | 86 | 1,425 | 1.37 |
| 1. National Research Center, Egypt | 7 | 98 | 1.53 | 101 | 940 | 1.05 |
| 1. Suez Canal University, Egypt | 14 | 88 | 1.08 | 93 | 831 | 1.16 |
| Countries | | | | | | |
| 1. Egypt | 306 | 3,321 | 1.39 | 2,425 | 19,457 | 1.04 |
| 1. South Africa | 109 | 933 | 0.95 | 273 | 2,810 | 1.04 |
| 1. Nigeria | 92 | 1,576 | 1.55 | 280 | 3,702 | 1.07 |
| 1. Tunisia | 22 | 132 | 0.76 | 123 | 520 | 0.45 |
| 1. Morocco | 16 | 92 | 0.49 | 119 | 706 | 0.70 |
| 1. Sudan | 21 | 99 | 0.41 | 102 | 694 | 0.73 |
| 1. Libya | 17 | 401 | 1.76 | 61 | 813 | 1.44 |
| 1. Ethiopia | 19 | 883 | 2.62 | 42 | 1,459 | 2.31 |
| 1. Ghana | 17 | 790 | 2.28 | 37 | 197 | 0.74 |
| 1. Kenya | 12 | 140 | 1.16 | 36 | 181 | 0.77 |
| Journals | | | | | | |
| 1. BMC Oral Health | 80 | 681 | 1.18 | 272 | 1,756 | 1.25 |
| 1. Ain Shams Dental Journal | 18 | 15 | 0.14 | 197 | 43 | 0.05 |
| 1. Journal of Contemporary Dental Practice | 9 | 20 | 0.78 | 101 | 315 | 0.36 |
| 1. Journal of Prosthetic Dentistry | 9 | 49 | 1.25 | 100 | 1,131 | 1.39 |
| 1. International Journal of Dentistry | 7 | 68 | 0.86 | 95 | 622 | 0.75 |
| 1. Brazilian Dental Science | 9 | 7 | 0.14 | 90 | 190 | 0.35 |
| 1. Clinical Oral Investigations | 11 | 200 | 2.67 | 85 | 784 | 2.03 |
| 1. Saudi Dental Journal | 14 | 118 | 0.92 | 54 | 483 | 0.92 |
| 1. Journal of International Oral Health | 7 | 4 | 0.10 | 59 | 76 | 0.23 |
| 1. Case Reports in Dentistry | 9 | 18 | 0.35 | 50 | 175 | 0.41 |

- 1. **Supplementary Table 3.** Research topic clusters, their prominence percentile, number of papers and their impact- all papers, papers addressing and not addressing SDGs

| Topic Cluster | Global  Prominence | All papers | | | With SDGs | | | With no SDGs | | |
| --- | --- | --- | --- | --- | --- | --- | --- | --- | --- | --- |
|  |  | Papers | PS (%) | FWCI | Papers | PS (%) | FWCI | Papers | PS (%) | FWCI |
| Implants | 89.73 | 785 | 1.42 | 0.87 | 18 | 0.03 | 1.23 | 767 | 1.39 | 0.86 |
| Scanning Electron Microscopy; Mechanical Strength & Zirconia | 79.99 | 659 | 1.85 | 1.22 | 60 | 0.17 | 1.98 | 599 | 1.68 | 1.15 |
| Endodontics & Dental Pulp | 74.69 | 637 | 1.74 | 1.15 | 24 | 0.07 | 0.8 | 612 | 1.68 | 1.17 |
| Fluoride; Oral Hygiene & Streptococcus mutans | 72.53 | 411 | 1.33 | 1.11 | 65 | 0.21 | 1.42 | 346 | 1.12 | 1.05 |
| Oral Hygiene; Gum (Oral Cavity) & Diabetes | 59.06 | 139 | 0.67 | 1.04 | 45 | 0.22 | 1.23 | 94 | 0.45 | 0.95 |
| Temporomandibular Joint; Masticatory Muscle & Thermography | 43.17 | 88 | 0.65 | 1.52 | 4 | 0.03 | 3.16 | 84 | 0.62 | 1.44 |
| Cleft; Orthodontics & Palate | 28.97 | 156 | 1.02 | 0.88 | 11 | 0.07 | 1.09 | 145 | 0.95 | 0.86 |

PS: Publication share

- 1. **Supplementary Table 4.** The top 10 cited papers with 1^st^ author affiliation in an African country

| Paper | Year | Number of authors | Citations | Type of paper | Affiliation of 1^st^ author, Country |
| --- | --- | --- | --- | --- | --- |
| 1. Mechanical properties of zirconia reinforced lithium silicate glass-ceramic. | 2016 | 2 | 284 | Article | Mansoura University, Egypt |
| 1. Staging and grading of oral squamous cell carcinoma: An update. § | 2020 | 11 | 185 | review | University of Misurata, Libya |
| 1. Ameloblastoma: current etiopathological concepts and management. § | 2018 | 4 | 165 | review | University of Lagos, Nigeria |
| 1. Self-etching ceramic primer versus hydrofluoric acid etching: Etching efficacy and bonding performance. | 2018 | 2 | 118 | Article | Suez Canal University, Egypt |
| 1. Diagnostic potential and future directions of biomarkers in gingival crevicular fluid and saliva of periodontal diseases: Review of the current evidence. | 2018 | 1 | 110 | review | Cairo University, Egypt |
| 1. Evaluation of corticotomy-facilitated orthodontics and piezocision in rapid canine retraction. | 2016 | 3 | 107 | Article | Ain Shams University, Egypt |
| 1. Oral Microbiome Signatures in Diabetes Mellitus and Periodontal Disease. | 2020 | 7 | 95 | Article | Cape Peninsula University of Technology, South Africa |
| 1. The effect of periodontal therapy on glycemic control and fasting plasma glucose level in type 2 diabetic patients: Systematic review and meta-analysis. § | 2016 | 2 | 95 | Article | University of Gondar, Ethiopia |
| 1. Influence of Age and Apical Diameter on the Success of Endodontic Regeneration Procedures. | 2016 | 4 | 92 | Article | Ain Shams University, Egypt |
| 1. Polymerization shrinkage assessment of dental resin composites: a literature review. | 2016 | 2 | 87 | Review | Cairo University, Egypt |

§: addressing SDGs

## Supplementary Figures


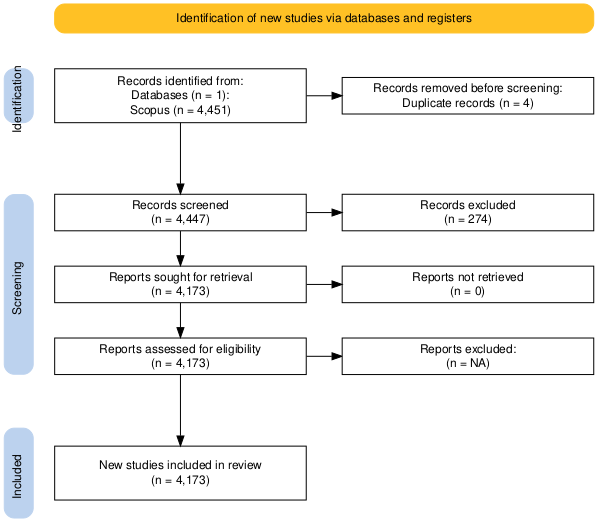


**Supplementary Figure 1.** Flow diagram^24^ of the number of papers included in the analysis.

**Supplementary Figure 2**. Top 10 publishers with the greatest number of published papers

|  |  |
| --- | --- |
|  |  |

**Supplementary Figure** 3. Most frequent 50 keywords in all papers by category
